# Gut Microbiota From Sjögren syndrome Patients Causes Decreased T Regulatory Cells in the Lymphoid Organs and Desiccation-Induced Corneal Barrier Disruption in Mice

**DOI:** 10.3389/fmed.2022.852918

**Published:** 2022-03-09

**Authors:** Laura Schaefer, Claudia M. Trujillo-Vargas, Firas S. Midani, Stephen C. Pflugfelder, Robert A. Britton, Cintia S. de Paiva

**Affiliations:** ^1^Center of Metagenomics and Microbiome Research, Department of Molecular Virology and Microbiology, Baylor College of Medicine, Houston, TX, United States; ^2^Department of Ophthalmology, Ocular Surface Center, Baylor College of Medicine, Cullen Eye Institute, Houston, TX, United States; ^3^Grupo de Inmunodeficiencias Primarias, Facultad de Medicina, Universidad de Antioquia UdeA, Medellin, Colombia

**Keywords:** Sjögren, dry eye, cornea, Tregs, microbiome

## Abstract

Sjögren syndrome (SS) is an autoimmune inflammatory disorder characterized by secretory dysfunction in the eye and mouth; in the eye, this results in tear film instability, reduced tear production, and corneal barrier disruption. A growing number of studies show that homeostasis of the ocular surface is impacted by the intestinal microbiome, and several 16S sequencing studies have demonstrated dysbiosis of the intestinal microbiota in SS patients. In this study, we utilized metagenomic sequencing to perform a deeper analysis of the intestinal microbiome using stools collected from sex- and age-matched healthy (*n* = 20), dry eye (*n* = 4) and SS (*n* = 7) subjects. The observed Operational Taxonomic Units (OTUs) and Shannon alpha diversity were significantly decreased in SS compared to healthy controls, and there was a significant inverse correlation between observed OTUs and ocular severity score. We also identified specific bacterial strains that are differentially modulated in SS vs. healthy subjects. To investigate if the differential composition of intestinal microbiome would have an impact on the immune and eye phenotype, we performed functional studies using germ-free mice colonized with human intestinal microbiota from SS patients and healthy controls. Flow cytometry analysis demonstrated reduced frequency of CD4^+^ FOXP3^+^ cells in ocular draining cervical lymph nodes (CLN) in mice colonized with SS patient intestinal microbiota 4 weeks post-colonization. We also found that offspring of SS-humanized mice also have fewer CD4^+^FOXP3^+^ cells in the CLN as well as spleen, demonstrating vertical transmission. SS-humanized mice subjected to desiccating stress exhibited greater corneal barrier disruption as compared to healthy control humanized mice under the same conditions. Taken together, these data support the hypothesis that the intestinal microbiota can modulate ocular surface health, possibly by influencing development of CD4^+^ FOXP3^+^ regulatory T cells (Tregs) in the ocular draining lymph nodes.

## Introduction

Sjögren syndrome (SS) is an autoimmune disorder affecting the secretory glands and mucosal tissues of the eye and mouth, including the lacrimal glands and salivary glands, and results in dry eye and dry mouth. A hallmark of this disease is lymphocyte infiltration of the lacrimal gland, salivary gland, and conjunctiva in the eye. Increased cytokine expression including IFNγ and IL-17 by the infiltrating lymphocytes and activated epithelial cells results in corneal barrier disruption and loss of goblet cells in the conjunctiva (1–4). A significant body of literature has linked the gut microbiome to development of disease and autoimmunity at distant sites, including the eye (5–9). We and others have previously shown that SS patients have reduced diversity of intestinal microbiota using 16S sequencing that in some studies correlates with more severe disease (10–14). In mouse models, germ-free mice lacking all bacteria spontaneously develop a SS-like syndrome that can be ameliorated by restoring the gut bacteria by fecal material transplant (FMT) (2, 8).

The commensal bacteria that inhabit the gastrointestinal tract are known to be instrumental in the modulation and maturation of the immune system and in the development of mucosal tolerance. It has been demonstrated in many studies that commensal bacteria can modulate the immune system via modulation of CD4^+^ CD25^+^ forkhead box protein 3 (FOXP3)+ regulatory T cells (Tregs) and IL-17-expressing immune cells (15–17). Autoimmune diseases including SS, uveitis, systemic lupus erythematosus, and rheumatoid arthritis are characterized by elevated levels of pro-inflammatory T cells including Th17 cells and reduced levels of anti-inflammatory Tregs (17–21). While bacteria can trigger autoimmune responses, certain commensal bacteria have also been shown to be protective and anti-inflammatory (7). In the eye, the balance of Th17 and Treg cells is a critical component of autoimmune uveitis with increased Th17 cells resulting in disease; proper balance can be restored by the commensal bacterial metabolite butyrate, ameliorating disease (17, 18). At the ocular surface, increased levels of IL-17-expressing T cells and decreased levels of FOXP3-expressing Tregs result in corneal barrier disruption and reduced numbers of goblet cells in the conjunctiva (3, 8), and butyrate treatment in mouse SS models can also improve disease.

In this study, we utilized metagenomic sequencing to perform a deeper analysis of the intestinal microbiome using stools collected from sex- and age-matched healthy, dry eye and SS subjects. Our results confirmed 16S sequencing to date in showing that decreased diversity in SS patients correlates with more severe disease. We identified specific bacterial species that were increased or decreased in the SS patient microbiome and correlated with disease severity. We demonstrated in mice that SS patient gut bacteria resulted in differential levels of Tregs, and this effect was passed onto offspring. In addition, mice colonized with SS bacteria exhibited greater corneal barrier disruption when exposed to desiccating stress.

## Materials and Methods

### Patient Evaluation and Sample Collection

The study design and execution conformed to the Declaration of Helsinki (22), and the protocol and the informed consent form were approved by the Baylor College of Medicine Institutional Review Board prior to study initiation. Female control subjects and patients with SS were enrolled in the study from January 2015 to January 2016 and from January 2019 to January 2021. In order to participate, patients gave written informed consent certifying that they understood the study purpose and possible consequences. SS patients were recruited from the multispecialty SS clinic at the Baylor College of Medicine (BCM) Alkek Eye Center and had a complete ocular, oral, and rheumatologic evaluation, including a panel of serum autoantibodies. All SS patients were diagnosed with primary Sjögren's syndrome without any other underlying autoimmune disorders and fulfilled the diagnostic criteria by the American College of Rheumatology/European League Against Rheumatism (23). Dry eye and healthy patients were evaluated in the BCM Alkek Eye Center using previously published aqueous tear-deficient dry eye and healthy subject criteria (24–26). All subjects selected for the study were female and age-matched, and included healthy (*n* = 20), dry eye (*n* = 4) and SS (*n* = 7) subjects. For clinical data measurements, the average of the left and right eye measurements was used. Symptom assessment in dry eye (SANDE) and Ocular Surface Disease Index (OSDI) symptom questionnaires, fluorescein tear break-up time (TBUT), Schirmer I test, cornea fluorescein and conjunctival lissamine green dye staining, and tear meniscus height measurement using optical coherence tomography (OCT) were performed as previously described (27). The ocular surface clinical parameters were all measured by the same observer (S.C.P.). Healthy control subjects had no eye irritation, a TBUT ≥ 7 s, Schirmer 1 ≥ 10 mm, corneal fluorescein score ≤ 2, conjunctival lissamine score ≤ 2, and no Meibomian gland disease. Subjects were excluded if they had prior laser-assisted *in situ* keratomileusis or corneal transplantation surgery, cataract surgery in the past year, punctal occlusion with plugs or cautery, a history of contact lens wear, use of topical medications other than preservative-free artificial tears, or chronic use of systemic medications known to reduce tear production. They were instructed not to instill any eye drops on the day of the evaluation. Ocular disease severity was scored following the guidelines published by the International Dry Eye Workshop (28). All SS KCS patients had a severity score ≥ 3, and healthy controls had a severity score = 0.

Patients were supplied with a stool collection kit with instructions and were asked to submit their samples within 24 h post-collection. Stool was collected using the Fisherbrand Commode Specimen Collection system (Fisher Scientific). The specimen container was sealed in a gallon sized Ziploc bag with two activated EZ Anaerobe Container System Sachets (BD Biosciences, New Jersey, USA). The sample was then sealed together with ice packs in a 4-gallon plastic container with a gasketed lid (Rubbermaid). Within 2 h of receipt, the container was transferred into an anaerobic chamber containing 5% CO_2_, 5% H_2_, and 90% N_2_, where the stool was divided into 12 g aliquots in 50 ml screwcap tubes for storage at −80°C. Fecal slurry was prepared for each patient sample by adding 36 ml anaerobic PBS to a 12 g aliquot, vortexing vigorously to emulsify, centrifuging at 200 g for 5 min, and aerobically transferring the supernatant into new tubes for storage at −80°C.

### Preparation of DNA for Metagenomic Sequencing

For each patient sample, DNA was extracted from 200 μl fecal slurry using a DNEasy Powersoil Pro kit (Qiagen, Maryland, USA). Samples were disrupted before extraction by homogenization in a Mini-Beadbeater-96 (Biospec Products, Oklahoma, USA) for 1 min; samples were then centrifuged at 8,000 g for 1 min and the supernatant was used for DNA extraction.

### Metagenomic Sequencing and Analysis

Metagenomic sequencing and sequence annotation was performed by Diversigen (Minneapolis, USA) with their BoosterShot Shotgun Sequencing service (https://www.diversigen.com/services/boostershot/). Raw sequence files were submitted to the Sequence Read Archive. Diversigen annotated the data by aligning DNA sequences to the CoreBiome Venti database, a curated proprietary database composed of genomes sourced from the NCBI Reference Sequence Database (RefSeq) plus additional manually curated strains; alignments were made at 97% identity against all reference genomes using fully gapped alignment with the BURST mapping algorithm (29). Filtered taxonomy tables were generated after exclusion of samples with <10,000 sequences and exclusion of OTUs accounting for less than one millionth of all strain-level markers and those with <0.01% of their unique genome regions covered (and <0.1% of the whole genome) at the species level. The number of counts for each OTU was normalized to the OTU's genome length. The normalized and filtered tables were used for all downstream analyses. Diversigen performed alpha diversity analysis including number of unique observed OTUs, Shannon diversity, and Chao richness calculations using a rarefied OTU table set to a 10,000 minimum depth using QIIME 1.9.1. Diversigen supplied data files including taxonomy, OTU, and functional analysis tables for further downstream analysis. The metagenomic dataset has been deposited in the NCBI Sequence Read Archive (BioProject ID PRJNA800662, www.ncbi.nlm.nih.gov/sra).

Statistical analysis was performed with GraphPad Prism 9.0 software (GraphPad Software Inc., San Diego, CA, USA) using one-way analysis of variance (ANOVA) with non-parametric Kruskal-Wallis tests for comparisons between multiple groups and with non-parametric Mann-Whitney U tests for pair-wise comparisons. *P* < 0.05 were considered significant. Statistical correlation analysis between disease severity score and alpha diversity measures was also made with GraphPad Prism. We performed beta diversity analysis and PERMANOVA (30) using the vegan package in R statistical software (31). Random Forest (32, 33) analysis was performed using the R randomForest package (34) to rank taxa based on their importance in discriminating healthy controls from SS patients. Differences in the relative abundances of these taxa between groups was further tested with two-sided Mann-Whitney and Kruskal-Wallis tests while correlation between the relative abundance of taxa and disease severity scores was tested with Spearman tests. Both tests were performed using the SciPy package version 1.7.1 in Python version 3.8.8 (35). Further, linear regression for severity scores on relative abundances was performed with the LinearRegression function in Scikit-learn version 0.24.2 in Python (36).

### Mice

Germ-free C57BL/6J mice were obtained from the Baylor College of Medicine Gnotobiotics Core Laboratory. For fecal material transplant (FMT), mice were colonized with human gut bacteria by oral gavage using 100 μl fecal slurry prepared with human patient stool. After gavage, mice were housed in a specific pathogen free barrier facility at Baylor College of Medicine in sterilized individually ventilated caging. Feed and drinking water were autoclaved. Animal studies were approved by the Institutional Animal Care and Use Committee at the Baylor College of Medicine. All studies adhered to the Association for Research in Vision and Ophthalmology Statement for Use of Animals in Ophthalmic and Visual Research. Sample size calculation was performed with StateMate Software version 2.0 (GraphPad Software Inc.) based on preliminary data. For flow cytometry experiments in colonized germ-free mice, two to nine female germ-free mice per patient sample from 8 different patient samples were gavaged with fecal slurry, for a total of 37 germ-free mice. Additional germ-free mice were gavaged with patient samples to establish breeding colonies; two to four germ-free breeder pairs were gavaged per patient sample to establish 6 colonies (3 SS and 3 healthy control colonies) for a total of 30 germ-free mice. For corneal barrier function studies, 11–14 female germ-free mice per patient sample for six patient samples were used for a total of 77 mice. For flow cytometry experiments in humanized offspring mice, 8–14 female mice per experimental group (experimental groups are healthy and SS) were sacrificed over the course of 3 experiments, for a total of 47 mice.

### 16S Sequencing and Analysis

DNA was isolated with Qiagen DNeasy Blood and Tissue kits as described previously (5). Bacterial 16S sequences spanning variable region V4 were amplified by PCR with primers F515/R806 and sequenced by Illumina MiSeq at the BCM Alkek Center for Metagenomics and Microbiome Research. 16S sequence data were processed using the MiSeq pipeline for mothur using software version 1.38.124,25 and the MiSeq SOP pipeline (version November 2021) (37) (http://www.mothur.org/wiki/MiSeq_SOP; in the public domain). Chimeric sequences were identified and removed using the mothur implementation of UCHIME. After classification with the mothur-formatted Silva reference files (version 132, https://www.arb-silva.de/silva-license-information), sequences classified as Eukarya, Archaea, chloroplast, mitochondria, or unknown were removed. Sequences present only once in the data set were also removed. Sequences were clustered from a distance matrix into operational taxonomic units (OTUs) with 97% similarity in mothur. 921 OTUs were identified across all samples with an average rarefaction depth of 24,200 reads per sample. The Agile Toolkit for Incisive Microbial Analyses (ATIMA) visualization toolkit developed by the Center for Metagenomics and Microbiome Research at Baylor College of Medicine (http://atima.jplab.net/; in the public domain) was used for data analysis. The 16S dataset has been deposited in the NCBI Sequence Read Archive (BioProject ID PRJNA805114, www.ncbi.nlm.nih.gov/sra).

### Flow Cytometry

Cervical lymph nodes and spleens were surgically collected and single-cell suspensions were prepared as described previously (5). 1 x 10^6^ cells/well were plated in a 96-well round bottom plate, incubated with 10 ng PMA (Sigma-Aldrich, St. Louis, MO, USA) and 1 μg ionomycin (Sigma-Aldrich) for 3 h at 37°C 5% CO_2_, then incubated for 2 h after addition of 1 ul GolgiStop (BD Biosciences). DNAse I was added to a final concentration of 3500 U/ml for the last 5 min of incubation. Cells were washed with FACS buffer (1% fetal bovine serum in PBS), resuspended in 100μl FACS buffer containing 0.5μl reconstituted live/dead near-IR fluorescent reactive dye (Invitrogen, Carlsbad, CA), and incubated for 10 min at 4°C. After washing with FACS buffer, cells were incubated with anti-CD16/32 (BD Pharmingen, San Diego, CA, USA) at 4°C for 10 min, followed by staining with anti-CD4_FITC (clone RM4-5, eBioscience, San Diego, CA, USA) and anti-CD45_Bv510 (clone 30-F11, Biolegend, San Diego, CA, USA). Cells were fixed and permeabilized according to manufacturer protocol with the BD Transcription Factor Buffer set (BD Biosciences). Cells were then stained with anti-FOXP3_APC (eBioscience). Fluorescence-minus-one controls were performed to define the gating strategy. Cell populations were gated on CD45^+^, CD4^+^, and then FOXP3^+^. Flow cytometry was performed on a BD Canto II Benchtop cytometer with BD Diva software version 6.7 (BD Biosciences). Final data were analyzed using FlowJo software version 10 (Tree Star Inc., Ashland, OR).

### Measurement of Corneal Barrier Function

Corneal barrier function was assessed by quantifying corneal epithelial permeability to 70-kDa Oregon Green Dextran-488 (OGD; Invitrogen, Carlsbad, CA) according to our previously published protocol (8). Images of stained corneas were taken with a high-resolution digital camera (Coolsnap HQ2, Photometrics, Tucson, AZ) attached to a stereoscopic zoom microscope (SMZ 1500; Nikon, Melville, NY). NIS Elements software (version 3.0, Nikon, Melville, NY) was used to grade the mean fluorescence intensity within a 2-mm diameter circle placed on the central cornea in the digital image; the scale is a continuous variable. Each image was quantified by two blinded observers. The mean intensity of the right and left eyes was averaged. Statistical significance for pairwise comparisons was assessed using one-way ANOVA and Mann-Whitney U tests using GraphPad Prism (GraphPad, Inc, version 9).

## Results

### Metagenomic Analysis Identifies Differential Bacterial Species Content and Decreased Diversity in SS Patient Stool

Twenty control subjects, four non-SS dry eye patients, and seven SS patients were evaluated in the BCM Alkek Eye Center (Table 1). SS patients were all diagnosed with primary Sjögren syndrome and did not have any other underlying autoimmune disorders. All patients completed the Ocular Surface Disease Index (OSDI) and Symptom Assessment In Dry Eye (SANDE) questionnaires to assess dry eye symptoms. Clinical signs were evaluated using several methods, including measurement of tear meniscus height (TMH), tear break-up time (TBUT), and corneal and conjunctival staining scores. The control subjects had minimal eye irritation symptoms and no clinical signs of keratoconjunctivitis sicca (KCS), while SS dry eye patients had significant symptoms and clinical signs. Statistical comparison of dry eye scoring metrics between controls, dry eye and SS KCS are shown in Table 2. Stool samples were collected and subjected to metagenomic sequencing analysis. Previous studies comparing SS gut microbiota to healthy human controls have used 16S sequencing to amplify the bacterial 16S rRNA gene with primers to conserved regions (10–14). One major advantage of metagenomic sequencing is that all DNA content in a sample is sequenced, so the resulting dataset is unbiased and not dependent on primers to detect and amplify a specific gene target. In this way, metagenomic sequencing is more sensitive and can identify bacterial taxa down to the species level.

**Table 1:** Demographic characteristics of study groups.

	**N, subjects**	**Age, mean, years**	**Age, range, years**	**Female** **/males**
Healthy	20	57	39–76	20/0
Non-SS DE	4	47	44–53	4/0
SS	7	59	40–70	7/0
*P*-value healthy vs. non-SS DE		NS		
*P*-value healthy vs. SS		NS		

*Statistical significance was assessed using non-parametric Mann-Whitney U-tests. DE, dry eye; SS, Sjögren syndrome*.

**Table 2:** Summary of clinical data.

	**OSDI score**	**SANDE score**	**Tear meniscus height in μm**	**Tear break-up time in seconds**	Corneal staining score^*^	**Conjunctival staining score^†^**
Healthy	9.9 ± 8.0	13.8 ± 18.4	415.4 ± 259.5	9.4 ± 2.3	0.1 ± 0.3	0.3 ± 0.7
Non-SS DE	38.5 ± 10.8	50.0 ± 25.2	213.5 ± 75.8	4.1 ± 1.3	1.4 ± 1.4	1.9 ± 1.5
SS	64.3 ± 11.2	77.0 ± 28.7	188.9 ± 119.4	2.7 ± 0.8	7.2 ± 2.9	5.3 ± 1.2
*P*-value healthy vs. non-SS DE	*P* < 0.001	*P* < 0.01	*P* < 0.05	*P* < 0.001	*P* < 0.01	*P* < 0.01
*P*-value healthy vs. SS	*P* < 0.0001	*P* < 0.0001	*P* < 0.0001	*P* < 0.0001	*P* < 0.0001	*P* < 0.0001

*Statistical significance was assessed using non-parametric Mann-Whitney U tests. OSDI, Ocular Surface Disease Index questionnaire; SANDE, Symptom Assessment iN Dry Eye (visual analog questionnaire); DE, dry eye; SS, Sjögren syndrome*.

* *Corneal fluorescein dye staining;*

† *Conjunctival lissamine green dye staining*.

**Figure 1:**
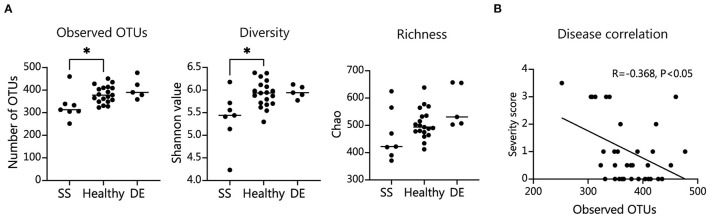
SS patient microbiome alpha diversity is significantly decreased and correlates with disease severity. **(A)** Number of observed operational taxonomic units (OTUs), Shannon diversity measure, and Chao richness in patient fecal samples. Statistical significance was assessed using non-parametric Mann-Whitney U tests. *P ≤ 0.05. **(B)** Spearman's correlation of combined severity index and number of intestinal OTUs; R, coefficient of correlation.

**Figure 2:**
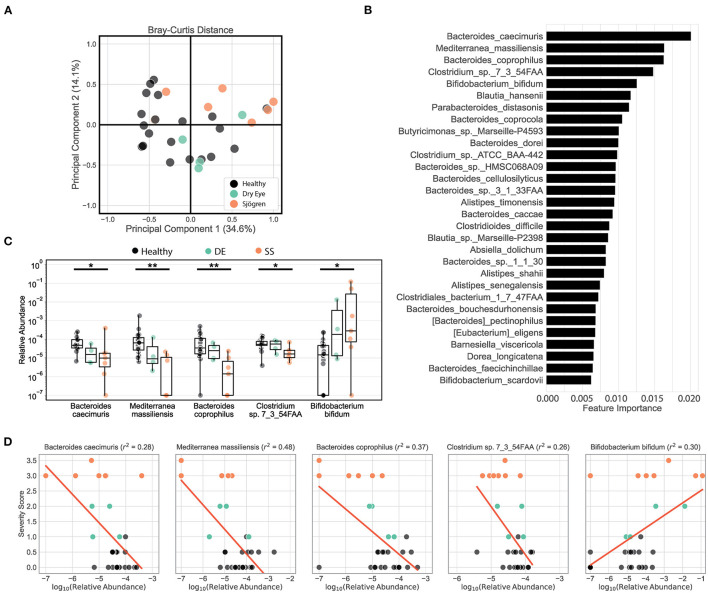
Metagenomic sequencing of SS patient stool shows decreased diversity and identifies differential bacterial species that correlate with disease severity. **(A)** Principal coordinate analysis of the Bray-Curtis distances between healthy, dry eye, and Sjögren syndrome (SS) subjects. **(B)** Top 25 species ranked based on their relative importance in a Random Forest model trained to separate healthy controls from SS patients. **(C)** Box plots describe the relative abundance in healthy controls, dry eye patients, and SS patients for the top 5 ranked species selected by the Random Forest model. Box plots show the median, the ranges between the first and the third quantile, and the ranges between the lowest and the highest value. Statistical significance was assessed using non-parametric Mann-Whitney U tests. *P ≤ 0.05, **P ≤ 0.01. **(D)** Scatter plots for the correlation between severity scores and log-transformed relative abundances for the top 5 species selected by the Random Forest model. Red lines show the predicted severity scores based on simple linear regression models. The goodness of fit is estimated by the coefficient of determination *r*^2^ shown in the title texts. For ease of visualization in scatter and box plots, a pseudocount of 10^−7^ was added to all relative abundances.

We tested our metagenomic data for group differences in within-subject diversity using Shannon diversity index, Chao richness, and the number of unique OTUs. Our analysis showed that SS microbial communities have significantly fewer observed OTUs, as well as significantly lower Shannon diversity (Figure 1A). Chao richness values were not significantly changed, although SS communities trended toward lower richness values. In addition, we observed that the number of observed OTUs negatively correlated with severity of disease as measured by the combined severity index (Spearman's ρ = −0.37, *P* = 0.03, Figure 1B). This agrees with our previously published results comparing a different group of SS patients to healthy controls using 16S sequencing to characterize their gut microbial communities (10). Using Bray-Curtis beta diversity analysis to investigate the between-subject differences between the patient groups, we found that there was significant separation between the SS patients and the healthy control group (PERMANOVA *R*^2^ = 0.08, *P* < 0.05), but not between the healthy and dry eye group. Figure 2A shows a principal coordinate analysis (PCoA) plot with the healthy, dry eye, and SS microbial communities. We next investigated the taxonomic differences driving the statistical separation between healthy controls and SS communities using the Random Forest machine learning algorithm. The Random Forest algorithm ranked taxa based on their importance in separating healthy from SS subjects which we confirmed by testing for differential abundance of each taxa using Mann-Whitney U tests. At the phylum level, Bacteroidetes (*P* < 0.05) and Actinobacteria (*P* < 0.01) contributed the most to community differences. At the species level, we identified several specific bacterial strains that collectively distinguished SS from healthy subjects (Figure 2B). For the top 5 ranked species, *Bacteroides caecimuris, Mediterranea masilliensis, Bacteroides coprophilus* and *Clostridium_sp_7_3_54FAA* were significantly decreased and *Bifidobacterium bifidum* was significantly increased in SS compared to healthy controls (all *P* < 0.01), illustrated in Figure 2C. These species also correlated with disease severity; more severe disease was positively correlated with decreased *Bacteroides caecimuris, Mediterranea masilliensis, Bacteroides coprophilus* and *Clostridium_sp_7_3_54FAA* and increased *Bifidobacterium bifidum* (Figure 2D). A full list is presented in Supplementary Table 1.

Taken together, our metagenomic data confirm results from 16S sequencing showing that the SS gut microbiome is less diverse, and reduced diversity correlates with disease severity. In addition, we have identified specific species that are either depleted or enriched in SS patients which correlate with disease severity.

### Mice Humanized With SS Fecal Material Exhibit Fewer FOXP3^+^ CD4^+^ Cells in Cervical Lymph Nodes (CLN) Than Mice Humanized With Normal Healthy Fecal Material

**Figure 3:**
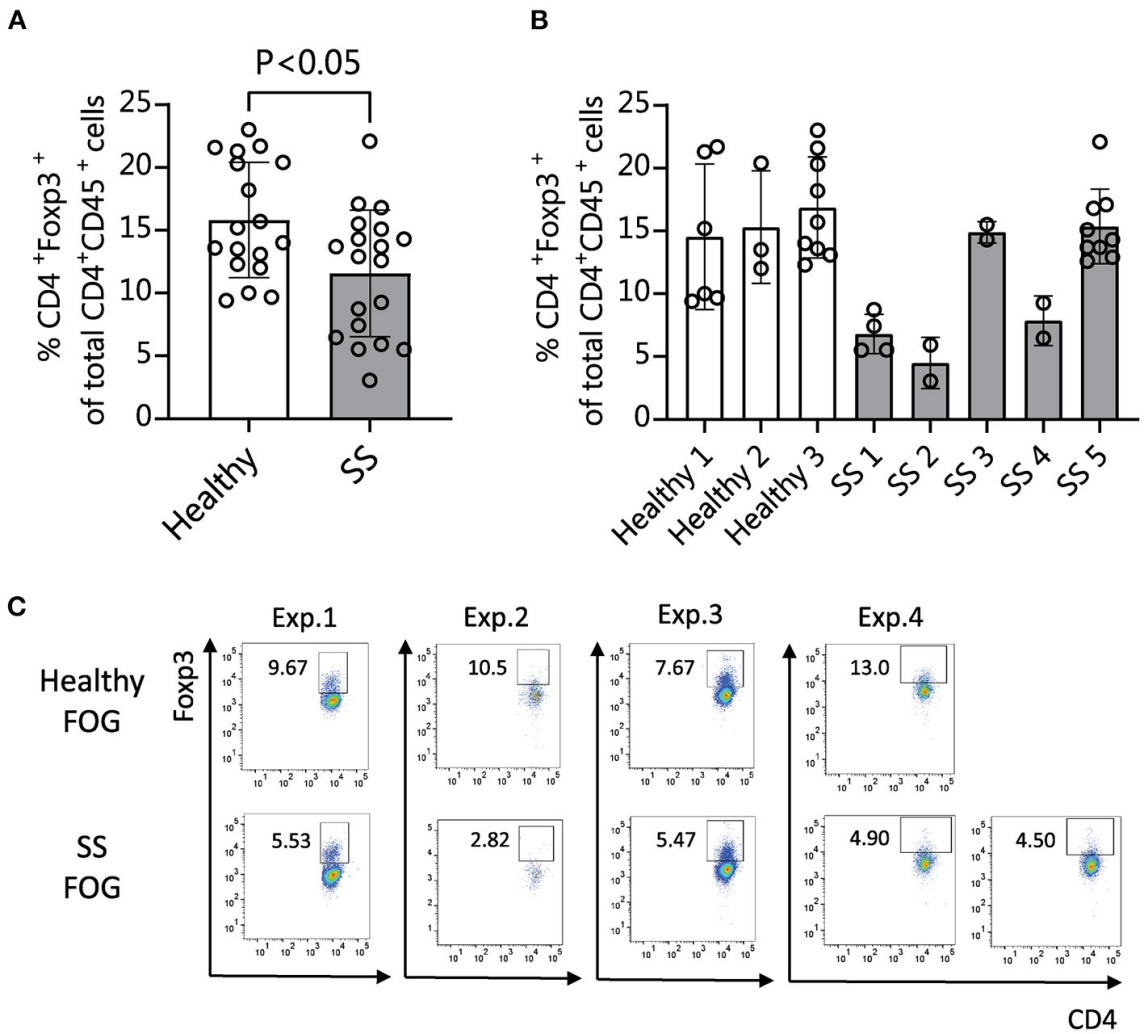
Mice colonized with SS fecal material exhibit fewer CD4^+^ FOXP3^+^ cells in cervical lymph nodes (CLN) than mice humanized with normal healthy fecal material. **(A,B)** 4 weeks after humanization of germ-free mice with patient stools, CLNs were collected for flow cytometry analysis and analyzed for the percentage of CD45^+^ CD4^+^ cells expressing FOXP3. **(A)** Shows the combined data for animals humanized with healthy vs. SS patient stools (*n* = 3–5 donors per group). **(B)** Shows the same data separated by patient donor. **(C)** Representative flow cytometry data showing CD45^+^ CD4^+^ FOXP3^+^ cell populations in 4 separate experiments using fecal slurries from 5 different SS patients and 4 healthy controls. Exp., Experiment; FOG, fecal oral gavage; SS, Sjögren syndrome.

We and others have shown that human patients with SS dry eye and non-SS dry eye have gut dysbiosis (10–14). To investigate if the different composition of intestinal microbiome between SS and heathy subjects would have an impact on the immune and eye phenotype, we performed functional studies using fecal material transplant (FMT) of human intestinal microbiota into germ-free C57BL/6J mice. FMT is used to colonize germ-free mice with a specific set of bacterial communities, since no other organism are present in the gut. Because dry eye is more frequent in women (38, 39), and male mice are resistant to corneal barrier disruption after desiccation or lacrimal gland excision (2, 40), only female mice were used. Four-week-old female germ-free C57BL/6J mice were gavaged with fecal material from either healthy donors or SS patients (*n* = 3–5 donors per group). We used multiple patient donors for each treatment group in order to account for biological variability between patients. The fecal material used for gavage and stools from a subset of recipient mice were submitted for 16S sequencing analysis. The alpha diversity of microbiota from SS stool recipient mice was lower than alpha diversity of microbiota from healthy stool recipient mice, recapitulating the difference observed in the original human samples (Supplementary Figure 1A). The taxonomic profiles from each group of mice gavaged with a particular patient sample look similar to each other, but were distinct from mice gavaged with other patient samples, indicating that each patient donor stool resulted in a distinct microbial community in the mouse gut and that the dysbiosis seen in the human patients was transferred to the mice (Supplementary Figure 1B). It has been shown that intestinal microbiota can modulate Tregs, which are critical in maintaining intestinal homeostasis (41, 42). Alterations in the frequency and function of Tregs in peripheral blood have been implicated in SS (19–21). Therefore, we investigated if the frequency of Treg cells was modulated by the intestinal microbiome. We collected immune cells from ocular-draining cervical lymph nodes (CLNs), spleens, intestinal lamina propria and mesenteric lymph nodes (MLN) 4 weeks post-microbiota humanization and performed flow cytometry analysis using CD45, CD4, and FOXP3 coexpression as markers for Treg cells. Mice colonized with SS patient fecal microbiota showed significantly decreased levels of CD45^+^ CD4^+^ FOXP3^+^ cells in CLN tissue compared with mice colonized with healthy control microbiota (Figure 3). No significant difference was observed in spleen, MLN, or lamina propria (not shown).

### Mice Humanized With SS Fecal Material Had Increased Corneal Barrier Disruption Compared With Mice Humanized With Fecal Material From Healthy Patients After Desiccation

**Figure 4:**
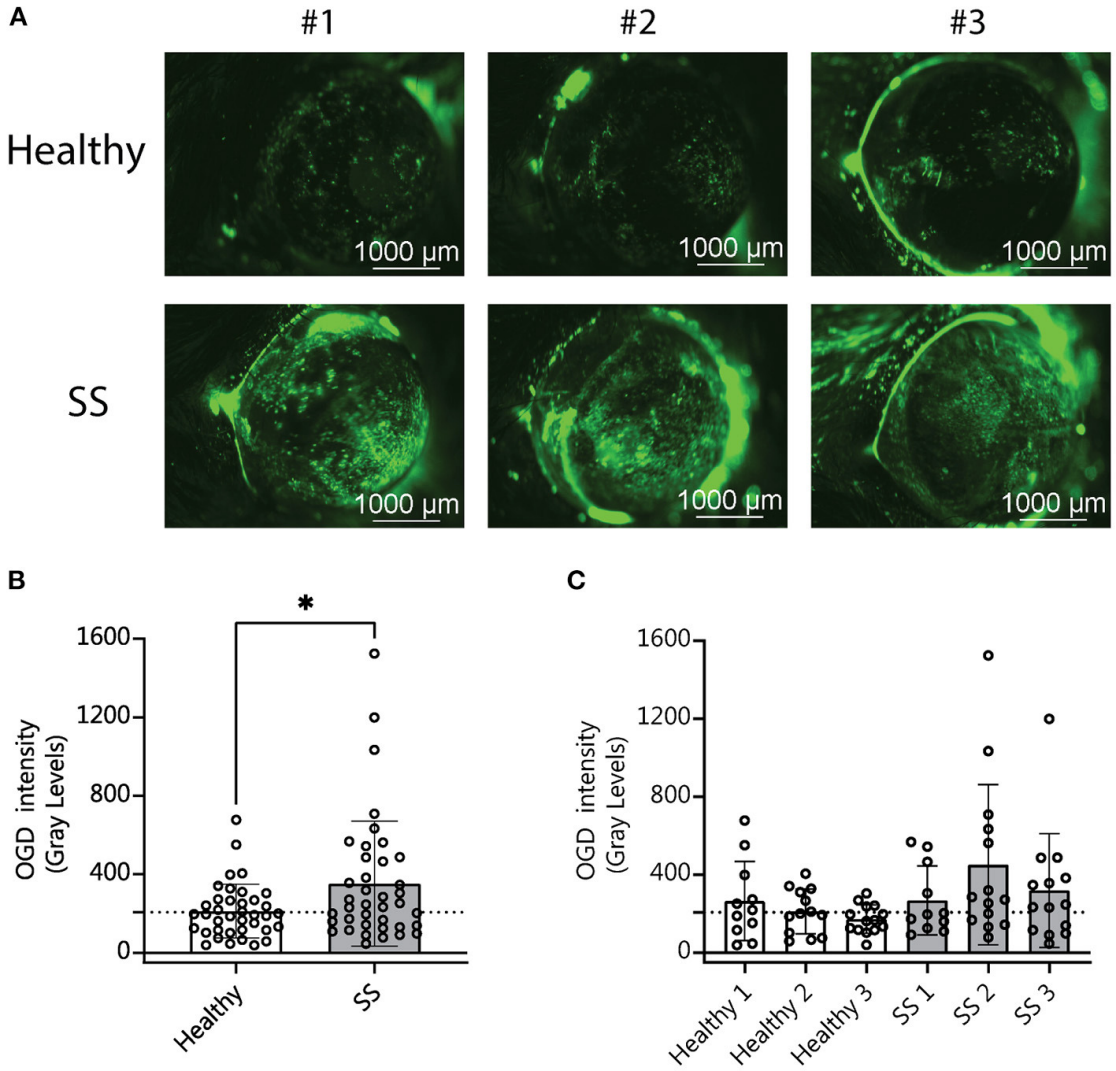
Mice colonized with SS fecal material had increased corneal barrier disruption after desiccating stress compared with mice colonized with fecal material from healthy patients. **(A)** Representative images of corneal permeability for animals humanized with either healthy or SS patient stool. Corneal barrier integrity was assessed by measuring the epithelial permeability to Oregon-green-dextran dye (OGD). **(B,C)** Cumulative OGD intensity measurement data from humanized mice subjected to 5 days of DS. Each data point represents the intensity value for one animal (averaged over the two eyes). **(B)** Shows the combined data for animals humanized with healthy vs. SS patient stools (*n* = 3 donors per group). **(C)** Shows the same data separated by patient donor. Statistical significance was assessed using non-parametric Mann-Whitney U-tests. *P ≤ 0.05.

Our analysis of the metagenomic data revealed that more severe disease correlates with aspects of the SS gut microbiota in humans, including less diversity and the presence or absence of specific bacterial taxa. We have also shown in previously published studies that dysbiosis worsens the dry eye phenotype in mice subjected to desiccating stress (DS) (10). We therefore asked if SS-humanized mice would have more severe disease in response to DS. Humanized mice (*n* = 3 different donors per group) were subjected to DS for 5 days according to published protocols (10). Uptake of Oregon-Green-Dextran (OGD) dye was used to evaluate corneal barrier function after DS. Mice colonized with SS patient gut bacteria exhibited decreased corneal barrier integrity as compared to mice colonized with healthy control bacteria (Figure 4). This indicates that a dysbiotic microbiome decreases the response to environmental stress, such as desiccation.

### Offspring of SS-Humanized Mice Also Have Fewer CD4^+^ FOXP3^+^ Cells in the CLN as Well as Spleen, Demonstrating Vertical Transmission

**Figure 5:**
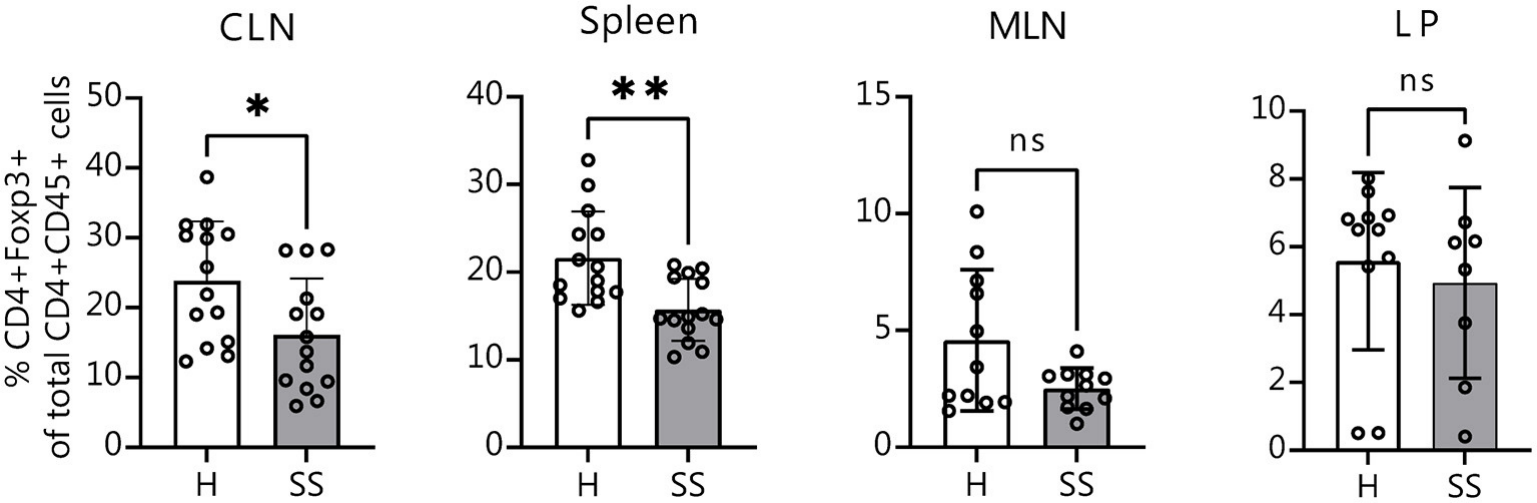
Offspring of SS-humanized mice also have fewer CD4^+^ FOXP3^+^ cells in the CLN as well as spleen, demonstrating vertical transmission. Cumulative flow cytometry data showing percentage of CD4^+^ FOXP3^+^ cells from CLN, spleen, MLN, and lamina propria of 8-week-old offspring of mice gavaged with patient stools. CLN, cervical draining lymph nodes; MLN, mesenchymal lymph nodes; LP, lamina propria. Statistical significance was assessed using non-parametric Mann-Whitney U-tests. *P ≤ 0.05, **P ≤ 0.01, ns, not significant.

It is known that in the absence of bacteria, immune cell development and maturation is aberrant in germ-free mice (42). We wanted to investigate if mice that are colonized from birth with SS or healthy control human gut bacteria would also show differences in immune populations. Eight-week-old offspring of humanized mice were sacrificed and CLNs, spleens, intestinal lamina propria and MLNs were collected for flow cytometry analysis as before. Offspring of mice colonized with SS patient gut bacteria exhibited fewer CD4^+^ FOXP3^+^ cells in CLN tissue and in spleen as compared with healthy control-colonized mice (Figure 5). This demonstrates that the modulation of Treg development by the gut microbiota can be vertically transferred from parent to offspring.

## Discussion

This study adds to a growing body of knowledge on the role of the gut microbiome in autoimmune diseases in general and in SS specifically. Our work supports the hypothesis that a dysbiotic microbial community in the gut contributes toward the pathogenesis of SS, with specific microbes or groups of microbes associated with eye disease severity. In agreement with previously published results using 16S sequencing (10, 13), we observed a decrease in the diversity of SS patient gut microbiota relative to healthy controls that correlates with ocular disease severity. Decreased microbial gut diversity has been observed in other autoimmune diseases including systemic lupus erythematosus (SLE) and rheumatoid arthritis (43–45). We observed that the decrease in diversity is accompanied by shifts in the composition of the community, with depletion of specific microbes that may be protective of the ocular surface, including several *Bacteroides* species. Commensal bacteria residing in the gut and eye have been shown to be protective in murine eye disease models (2, 10, 46, 47). Depletion of commensal bacteria in both the DS mouse model and the CD25 knockout mouse model of dry eye resulted in more severe dacryoadenitis, and reconstitution of the microbiota ameliorated disease (2, 8). In another study, depletion of gut commensals resulted in decreased levels of immune effectors in the tear film and a weakened ocular surface barrier making it more susceptible to *Pseudomonas aeruginosa*–induced keratitis; mono-colonization of germ-free mice with a specific species, *Bacteroides acidifaciens*, restored ocular surface secretory IgA levels (48). Conversely, the SS gut microbiome is enriched for certain bacterial taxa, such as *B. bifidum* in this study, which may be contributing toward the eye pathology observed in these SS patients. Detrimental effects of commensal bacteria have been observed in several inflammatory diseases. It is known that commensal bacteria can trigger autoimmune responses by producing proteins that mimic human antigens, including in autoimmune uveitis, SLE and rheumatoid arthritis (6, 49, 50). High fat diet-induced dysbiosis in mice worsened the corneal epithelium's ability to heal in response to injury; fecal transplant using stool from mice fed a normal diet led to normalization of wound closure rates (51). Our work is a step toward identification of specific taxa that could either protective of the ocular surface or pathogenic.

Unlike 16S sequencing, metagenomic analysis allows identification down to the species level, and our Random Forest analysis identified distinct species that were important in distinguishing our SS patient cohort from the healthy controls. Previously published studies using 16S sequencing have revealed genera that were either depleted or enriched in SS dry eye patients, but the genera identified as changed have varied from study to study (10–14). The control groups have also differed, and not all used age- and sex-matched controls as we have in our current study. Among the most important species driving the statistical separation between our SS and healthy subject cohorts were several *Bacteroides* members, which were reduced in SS patients, and *Bifidobacterium bifidum*, which was increased. A 16S study from our lab using a different set of patients than the ones in the current study also found an elevated abundance of *Bifidobacterium* and a decrease in *Bacteroides* in SS patients (10). However, a 16S study from another group that used age- and sex-matched controls found decreased levels of genus *Bifidobacterium* in their SS cohort (11). Depending on the environmental context and the specific strain, *Bifidobacterium* strains can induce differential expression of cytokine profiles in dendritic cells, resulting in polarization of differentiating T-cells toward more pro-inflammatory effector cells or toward more anti-inflammatory Tregs (52, 53). Interestingly, the same 16S study found decreased levels of *Alistipes*; we also observed decreased levels of several *Alistipes* species in our SS cohort (Supplementary Table 1). Decreased *Alistipes* has also been observed in psoriatic arthritis and Crohn's disease (54, 55). While our metagenomic analysis here and previously published microbiome analyses suggest associations between certain taxa and ocular surface health, more functional studies will be needed to determine if there are causative links. Our functional studies in mice show that there is a causative link between the SS microbiota and the ocular surface. Whether any of the species identified in our metagenomic analysis is responsible for these functional differences is being addressed in ongoing studies in our lab.

Indeed we show in a mouse model that there are functional differences in the SS gut microbiota that impact ocular surface health. Colonization with gut commensals from SS patients resulted in differences in immune cell populations in eye-associated lymphoid tissue, with decreased numbers of Tregs. Under DS, this translated to loss of function of the corneal barrier. This suggests that bacteria residing in the gut can exert a protective effect by their presence in a healthy gut, a pathogenic effect by their presence in a dysbiotic gut, or both. The mechanism and identification of the taxa responsible are being addressed in ongoing studies. The mechanisms behind a protective effect of a healthy microbiota may be a direct effect such as a metabolite produced by the microbiota that is transported to the ocular surface via the vascular system. Alternatively it may be an indirect effect, with the microbiota influencing the development of immune cells that act at the ocular surface. There is evidence for both of these scenarios. For example, microbial-derived TLR2 ligands induce Treg proliferation (56). Also, polysaccharide A from the commensal *Bacteroides fragilis* facilitates the tolerogenic function of Tregs in the mucosa in a TLR2-dependent manner (57). There is a large body of work on commensal bacteria-produced short chain fatty acids (SCFAs) such as butyrate and their ability to modulate host gene expression and interact with host signaling pathways. SCFAs produced in the gut have been shown to have anti-inflammatory effects that extend beyond the gastrointestinal tract to distal sites in the body, including the eye (18, 58–60). Butyrate produced in the gut has been shown to impact inflammation indirectly at distal sites by increasing the levels of peripheral anti-inflammatory Tregs (18, 59, 61, 62). Butyrate originating in the gut has also been shown to be protective of the ocular surface under DS conditions. It is possible that the depletion of taxa in SS patients that normally produce anti-inflammatory compounds such as butyrate result in worse dry eye pathology.

The gut commensal bacteria play a critical role in education of the host immune system and in modulation of immune responses; bacterial metabolites such as SCFAs may be just one mechanism for how the microbiota exerts its effects. We have previously shown that Treg cells are protective at the ocular surface (1, 8). It has also been shown that DS induces dysfunctional regulatory T cells (63). FOXP3^+^ Tregs are significantly decreased in colonic lamina propria of germ-free and antibiotic-treated B6 mice; reconstitution of the microbiota by fecal transplant restores the normal frequency of Foxp3+Tregs, indicating that signals from microbiota modulate Treg numbers in the gut (64). The ability of the gut microbiota to modulate the balance between anti-inflammatory Treg and inflammatory Th17 cells has been demonstrated in *in vitro* and *in vivo* models of autoimmune disease (16, 17).

In this study, we identified differences in the taxonomic composition of gut microbial communities from SS and healthy patients down to the species level, that correlate with disease severity. We showed that colonization of germ-free mice with these SS bacterial communities resulted in decreased levels of Treg cells in the eye-draining lymph nodes. In addition, when the colonized mice are subjected to DS, SS-colonized mice have worse corneal barrier function than mice colonized with healthy control microbiota, demonstrating that the bacterial communities from SS patients are less protective of the ocular surface.

## Data Availability Statement

The datasets presented in this study can be found in online repositories. The metagenomic dataset has been deposited in the NCBI Sequence Read Archive (BioProject ID PRJNA800662, www.ncbi.nlm.nih.gov/sra). The 16S dataset has been deposited in the NCBI Sequence Read Archive (BioProject ID PRJNA805114, www.ncbi.nlm.nih.gov/sra).

## Ethics Statement

The studies involving human participants were reviewed and approved by Institutional Review Board at Baylor College of Medicine. The patients/participants provided their written informed consent to participate in this study. The animal study was reviewed and approved by Institutional Animal Care and Use Committee at the Baylor College of Medicine.

## Author Contributions

LS, CT-V, and CP: conceptualization and methodology. LS, CT-V, and FM: formal analysis. CP and RB: funding acquisition. CP: project administration and supervision. LS: writing—original draft. LS, CT-V, FM, SP, RB, and CP: writing—review and editing. All authors contributed to the article and approved the submitted version.

## Funding

This work was supported by the NIH/NEI EY026893 (CP), NIH EY002520 (Core Grant for Vision Research Department of Ophthalmology), Research to Prevent Blindness Stein Innovation Award (RB), Research to Prevent Blindness (Department of Ophthalmology), the Hamill Foundation, the Sid Richardson Foundation, and by Baylor Cytometry and Cell Sorting Core (CPRIT Core Facility Support Award [CPRIT-RP180672], the NIH [CA125123 and RR024574]). CT-V received supplemental salary support from Facultad de Medicina, Universidad de Antioquia, UdeA, Medellin, Colombia.

## Conflict of Interest

The authors declare that the research was conducted in the absence of any commercial or financial relationships that could be construed as a potential conflict of interest.

## Publisher's Note

All claims expressed in this article are solely those of the authors and do not necessarily represent those of their affiliated organizations, or those of the publisher, the editors and the reviewers. Any product that may be evaluated in this article, or claim that may be made by its manufacturer, is not guaranteed or endorsed by the publisher.

## Supplementary Material

Supplementary Figure 1 Germ-free mice colonized with human fecal material show similar gut microbial alpha diversity as human donor material with distinct taxonomic patterns. Human donor fecal material and fecal pellets from colonized mice were analyzed using 16S sequencing. **(A)** In both human and mouse samples, SS-derived samples showed lower alpha diversity measures: number of observed operational taxonomic units (OTUs), Shannon diversity, and Chao richness. Statistical significance was assessed using non-parametric Mann-Whitney U tests. ^*^P < 0.05, ^**^P < 0.01. **(B)** Bar graph showing taxonomic abundances from human donor fecal material (Hu) and from fecal pellets from 1 to 4 individual recipient mice (M1-M4). Taxa are shown at the Order taxonomic level.

Supplementary Table 1 Random Forest analysis of Healthy vs. SS subjects. Table lists all species included in the Random Forest models either for discriminating healthy controls from SS subjects (“Healthy vs. SS” model) or regressing severity score on relative abundance of bacterial taxa (“Severity Score” model). Species are described by their relative feature importance's and importance ranks for each of those models. In addition, species are described by their median relative abundance in each of the Healthy, Dry Eye (DE), and SS groups. Table also includes results for statistical testing including the two-sided non-parametric Mann-Whitney U statistic and *P*-value for comparing healthy controls and SS patients, and the Kruskal-Wallis H Statistic and *P*-value for comparing healthy controls, DE, and SS patients. Finally, table includes the Spearman's ρ statistic and *P*-value for the correlation between disease severity scores and the relative abundance of each species. For this analysis, we did not add any pseudocounts to species relative abundances prior to statistical testing.
