# Supplementary figures and images for: Gut Microbiota From Sjögren syndrome Patients Causes Decreased T Regulatory Cells in the Lymphoid Organs and Desiccation-Induced Corneal Barrier Disruption in Mice
Source: Front Med (Lausanne). 2022 Mar 9;9:852918. doi: 10.3389/fmed.2022.852918 (PMC8959809; doi:10.3389/fmed.2022.852918)

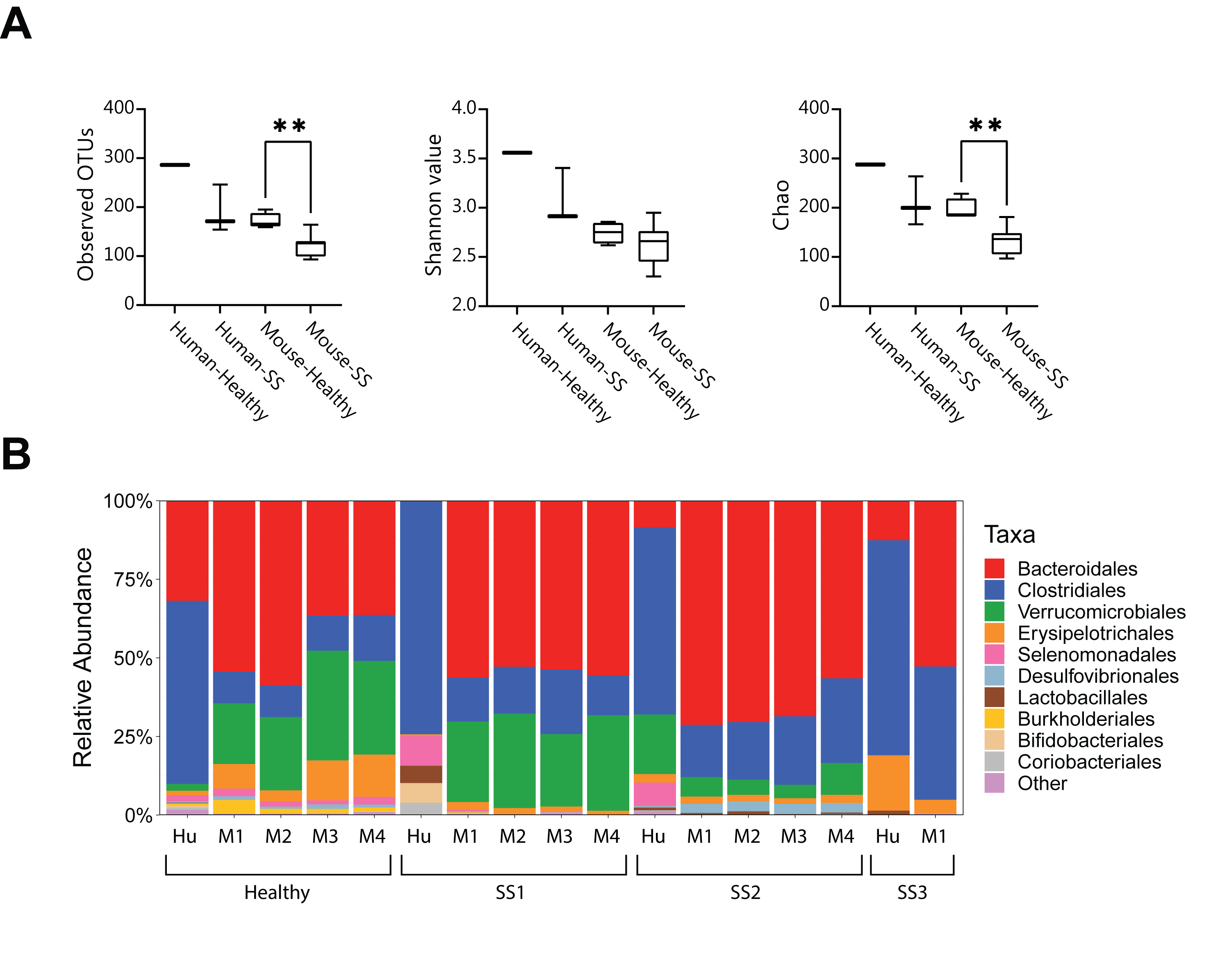

Supplement: Supplementary Figure 1 — Germ-free mice colonized with human fecal material show similar gut microbial alpha diversity as human donor material with distinct taxonomic patterns. Human donor fecal material and fecal pellets from colonized mice were analyzed using 16S sequencing. (A) In both human and mouse samples, SS-derived samples showed lower alpha diversity measures: number of observed operational taxonomic units (OTUs), Shannon diversity, and Chao richness. Statistical significance was assessed using non-parametric Mann-Whitney U tests. *P < 0.05, **P < 0.01. (B) Bar graph showing taxonomic abundances from human donor fecal material (Hu) and from fecal pellets from 1 to 4 individual recipient mice (M1-M4). Taxa are shown at the Order taxonomic level. [file Image_1.tif]
